# Cost-utility analysis of atezolizumab combined with bevacizumab for unresectable hepatocellular carcinoma in Thailand

**DOI:** 10.1371/journal.pone.0300327

**Published:** 2024-03-21

**Authors:** Supachaya Sriphoosanaphan, Witthawat Pantumongkol, Wantanee Kulpeng, Chanchai Charonpongsuntorn, Tawesak Tanwandee, Wattana Sukeepaisarnjaroen, Abhasnee Sobhonslidsuk, Pisit Tangkijvanich

**Affiliations:** 1 Department of Medicine, Division of Gastroenterology, Faculty of Medicine, Chulalongkorn University, Bangkok, Thailand; 2 Center for Value Driven Care, Faculty of Medicine Siriraj Hospital, Mahidol University, Bangkok, Thailand; 3 Center of Excellence in Hepatitis and Liver Cancer, Faculty of Medicine, Chulalongkorn University, Bangkok, Thailand; 4 Department of Internal Medicine, Medical Oncology Unit, Srinakharinwirot University, Nakhon Nayok, Thailand; 5 Division of Gastroenterology, Faculty of Medicine Siriraj Hospital, Mahidol University, Bangkok, Thailand; 6 Department of Medicine, Gastroenterology Unit, Srinagarind Hospital, Faculty of Medicine, Khon Kaen University, Khon Kaen, Thailand; 7 Department of Medicine, Division of Gastroenterology and Hepatology, Faculty of Medicine, Ramathibodi Hospital, Mahidol University, Bangkok, Thailand; Hokkaido University: Hokkaido Daigaku, JAPAN

## Abstract

**Background:**

Clinical trials have proven the efficacy and safety of atezolizumab combined with bevacizumab (A+B) in treating unresectable hepatocellular carcinoma (uHCC). This study aimed to assess the cost-utility of A+B compared to best supportive care (BSC) among uHCC patients in Thailand.

**Methods:**

We conducted a cost-utility analysis from a societal perspective. We used a three-state Markov model to estimate relevant costs and health outcomes over the lifetime horizon. Local cost and utility data from Thai patients were applied. All costs were adjusted to 2023 values using the consumer price index. We reported results as incremental cost-effectiveness ratios (ICERs) in United States dollars ($) per quality-adjusted life year (QALY) gained. We discounted future costs and outcomes at 3% per annum. We then performed one-way sensitivity analysis and probabilistic sensitivity analysis to assess parameter uncertainty. The budget impact was conducted to estimate the financial burden from the governmental perspective over a five-year period.

**Results:**

Compared to BSC, A+B provided a better health benefit with 0.8309 QALY gained at an incremental lifetime cost of $45,357. The ICER was $54,589 per QALY gained. The result was sensitive to the hazard ratios for the overall survival and progression-free survival of A+B. At the current Thai willingness-to-pay (WTP) threshold of $4,678 per QALY gained, the ICER of A+B remained above the threshold. The projected budgetary requirements for implementing A+B in the respective first and fifth years would range from 8.2 to 27.9 million USD.

**Conclusion:**

Although A+B yielded the highest clinical benefit compared with BSC for the treatment of uHCC patients, A+B is not cost-effective in Thailand at the current price and poses budgetary challenges.

## Introduction

Liver and intrahepatic bile ducts cancer (ICD-10-TM code C22.0) is the sixth most common cancer in the world [1]. In 2018, there were more than 800,000 new cases of liver and bile duct cancer worldwide. In Thailand, the age-standardized incidence of liver cancer is 21 cases per 100,000 population, making it the second most common type of cancer after breast cancer, according to the World Health Organization’s criteria. Hepatocellular carcinoma (HCC) is a common form of primary liver cancer and the leading cause of cancer-related death. The prognosis of HCC is poor, with the mortality rate of 20.9 deaths per 100,000 population, twice that of breast cancer.

HCC frequently presents in individuals within the age range of 40 to 70 years [2], with a notable likelihood of recurrence and metastasis [3]. Retrospective reviews in Japan [4] and the United States [5], demonstrated that 47–53 percent of HCC metastasize to the lungs. Patients with metastatic HCC had a median survival of approximately 6.9 months [6] and a median survival period without treatment of approximately 62 days (interquartile range (IQR) 31–153 days) [7].

The management of HCC is intricate. Multiple factors are considered in determining treatment selection for individuals such as tumor staging, liver function, and patient’s performance status [8]. In early-stage HCC patients, cure potential exists through surgery or liver transplantation. Nonetheless, a proportion of patients eventually present metastatic or advanced disease [9]. Current clinical practice guidelines recommend systemic therapy for patients with advanced or unresectable HCC (uHCC). First-line treatments include oral tyrosine kinase inhibitor i.e., sorafenib and lenvatinib, as well as newest regimens, i.e., a combination of atezolizumab and bevacizumab (henceforth, “A+B”) [10]. A Phase-III clinical trial of A+B known as IMbrave150 reported that A+B improved survival among uHCC patients compared to controls who received sorafenib (hazard ratio (HR) 0.66 [95% confidence interval (CI), 0.52, 0.85]; P = 0.0009), with the overall survival of 19.2 months [11].

In Thailand, there exists a significant unmet need for systemic therapy drugs in the treatment of uHCC, as none of these medicines are included in the National List of Essential Medicines. Within the context of current healthcare practice in Thailand, HCC patients who are covered under the Universal Coverage Scheme (UCS) and Social Security Scheme (SSS), representing the majority of Thai patients, receive best supportive care (BSC) when they are deemed ineligible for surgical treatment or loco-regional treatment options.

Although A+B has shown improvement in survival outcome and has the potential to serve as an alternative first-line treatment for uHCC, its cost presents a barrier to access. Cost-utility analysis (CUA) of A+B can inform clinicians and policymakers on the feasibility of allocating resources for public health to include the A+B regimen on Thailand’s National List of Essential Medicines and mitigate this barrier. However, there have been no studies assessing cost-utility of A+B compared with BSC and no studies conducted in the Thai setting. Therefore, the objective of this study is to present a cost-utility of A+B for Thai uHCC patients.

## Materials and methods

We conducted a model-based economic evaluation to estimate costs and treatment outcomes of ‘A+B’, an alternative treatment, compared to ‘the BSC’, the current standard treatment for uHCC patients under UCS and SSS in Thailand. We created a model of a hypothetical cohort of 50-year-old uHCC patients based on Thai expert opinions in the relevant fields. We adopted a societal perspective using a life-time horizon with 3% discounting for both costs and outcomes beyond a one-year period, as recommended by the Thai Health Technology Assessment guidelines [12, 13].

### Model structure and assumptions

**Fig 1:**
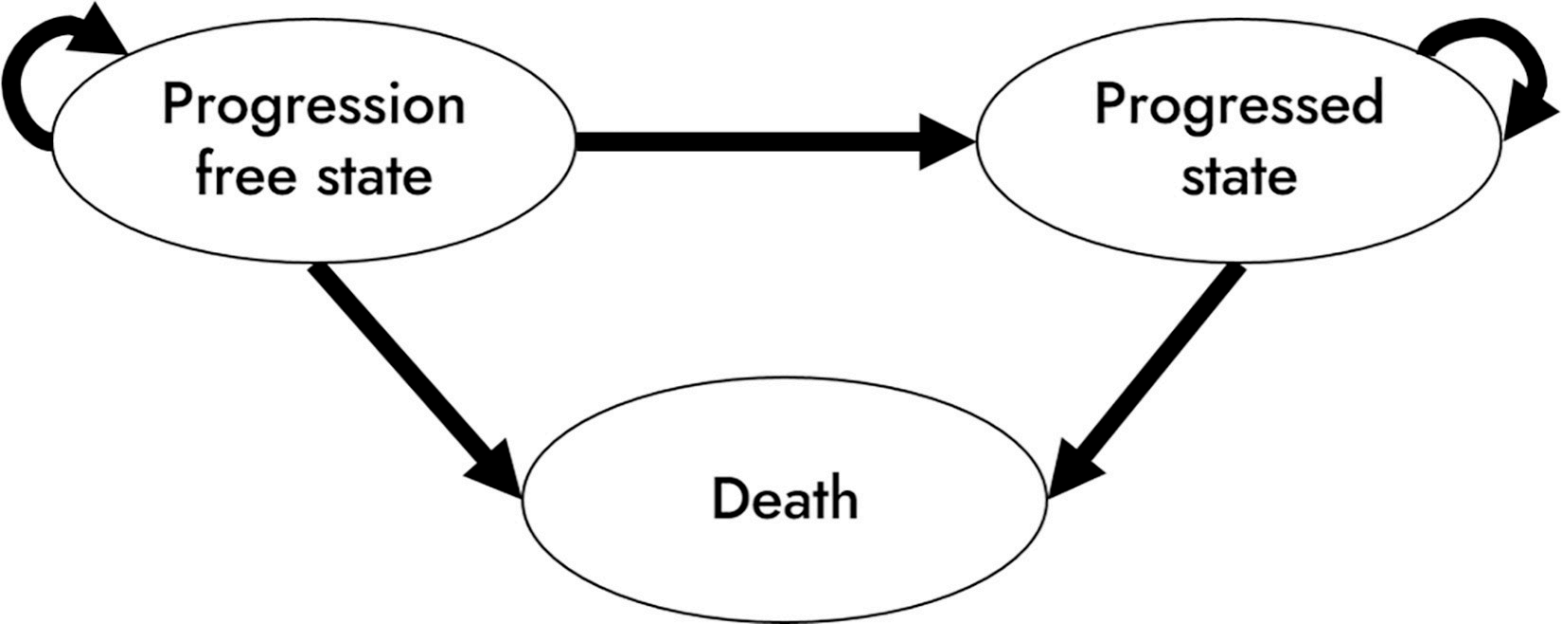
Markov model structure with the three health states.

We constructed a Markov model based on the progressive nature of uHCC (Fig 1). The model consisted of three major health states: (i) no progression, also known as the pre-progression state; (ii) progression, also known as the post-progression state, and; (iii) death. The cohort enters the model at the progression free state (i) and the arrows represent the transitional probabilities of moving from one health state to another (Fig 1). We deployed a one-month cycle in the model and assumed that patients would receive the allocated drug until the disease progressed.

### Model input parameters

#### Clinical parameter and transitional probability

We measured drug efficacy in terms of progression free survival (PFS) and overall survival (OS). However, there was no head-to-head trial comparing the combination of atezolizumab and bevacizumab with best supportive care. Therefore, a network meta-analysis was conducted to obtain efficacy parameters for this study. Prior to the indirect comparison, systematic review from RCTs examining atezolizumab and bevacizumab versus best supportive care or placebo were performed [14–16] (S1 Appendix). We measured the treatment effects using the hazard ratio (HR) (Table 1). We also estimated treatment costs for adverse reactions due to drug side effects according to reported clinical evidence [11]. Only the major grade 3 to 4 adverse effects (with an occurrence rate of more than 5%) are selected as agreed upon in the expert consultation meeting. This includes hypertension and increased aspartate aminotransferase (S1 Appendix).

**Table 1:** Input parameters used in the model.

Parameter	Distribution	Mean	SE	References
**Progression free survival**				
Hazard ratio for progression free survival of atezolizumab combined with bevacizumab compared vs BSC	Lognormal	0.34	0.06	Network Meta-analysis
**Probability of progression (per 1 month cycle)**				
Probability of progression in BSC: month 1–6	Fixed	0.29	N/A	[17]
Probability of progression in BSC: month 7 onward	Fixed	0.24	N/A	[17]
**Overall survival**				
Hazard ratio for overall survival of atezolizumab combined with bevacizumab vs BSC	Lognormal	0.40	0.07	Network Meta-analysis
**Probability of death from HCC (per 1 month cycle)**				
Probability of death in BSC: month 1–12	Fixed	0.17	N/A	[17]
Probability of death in BSC: month 13 onward	Fixed	0.10	N/A	[17]
**Direct medical cost**				
Best supportive care cost per month	Gamma	489	49	[18]
Atezolizumab combined with Bevacizumab cost per month*	Gamma	6,135	614	Reference price
Drug administration of Atezolizumab combined with Bevacizumab cost per month (Cost of cytotoxic and administrative)	Gamma	21	2	[19]
Unit cost of laboratory test**				
INR (Prothrombin Time)	Gamma	3	2.2	[19]
AFP Test	Gamma	12	7.4	[19]
Liver Function Test	Gamma	15	8.6	[19]
Complete blood count	Gamma	4	2.7	[19]
Unit cost of imaging test				
CT scan—upper abdominal^†^	Gamma	218	11.1	[19]
Chest scan without contrast^††^	Gamma	76	3.9	[19]
**Direct non-medical cost per month**				
Travel cost	Gamma	5	0.4	[19]
Additional food cost	Gamma	2	0.2	[19]
Productivity loss of caregiver	Gamma	3	1.2	[19]
**Utility**				
Utility of patients in progression free stage	Beta	0.89	0.02	Data from a local study^¶^ [20]
Utility of patients in progression stage	Beta	0.58	0.08	Data from a local study^¶^ [20]

**Abbreviation:** BSC, best supportive care; HCC, hepatocellular carcinoma; SE, standard error; USD, the United States dollar; vs, versus; *Mean body weight of 63 kg from the SizeThailand was used; **every month; ^†^every 2 months; ^††^every 3 months; ^¶^ We contacted the corresponding author of the referenced study to calculate mean utility data specific to our health states from their dataset.

We determined the probability of disease progression and death from HCC by employing hazard ratio (HR) for survival endpoint. Thai patients who received BSC served as the reference group (comparator) for comparison with A+B, and included data on PFS and OS. We converted PFS and OS data into 1-month cycle transitional probability using the following formula:

*Transitional probability per cycle* = *1-exp(-(-ln(1-P)/t))*,

*where P is OS or PFS at time T (month) and t is time T divided by 12*

We sourced data on probabilities of death from other causes from the data on mortality rate of the Thai general population stratified by age group [21] (S1 Appendix).

#### Costs and outcomes

We performed cost analysis based on a societal perspective, and included both direct medical and direct non-medical costs (Table 1). Direct medical cost included cost of requiring A+B drug, cost of grade 3/4 AE management (S1 Appendix), cost of necessary test for monitoring, and cost of BSC. Unit costs were sourced from the Drug and Medical Supply Information Center (DMSIC), Thailand FDA and the Thai standard cost list [19]. While, the resource utilization is obtained from HCC experts. The cost of BSC was derived from a study in Thailand [18], which included costs related to palliative care and conventional chemotherapy. We derived direct non-medical costs, such as costs for transportation, meals, and productivity loss by caregivers for hospital visits from the Thai standard cost list [19]. Monthly hospital visit was applied. All cost parameters are presented in 2023 the United States Dollar ($) (exchange rate: 34 Thai Baht = $1). We used the consumer price index (CPI) from the Bureau of Trade and Economic Indices to convert costs from other years to those for the year 2023 [22]. Outcomes were measured in terms of quality-adjusted life years (QALY) (Table 1). Utility data for each health state was derived from a prospective observational study of first-line systemic therapy in Thai advanced HCC patients [20].

### Data analysis

#### Incremental cost-effectiveness ratio

We conducted a cost-utility analysis to compare between the treatment options, and presented results as incremental cost-effectiveness ratio (ICER) per QALY gained. The ICER was then interpreted according to decision makers’ willingness-to-pay (WTP) or cost-effectiveness threshold. In the Thai healthcare setting, a given technology or medicine would be considered as a cost-effective option if the ICER was less than the threshold of $4,678 per QALY gained [12].

#### Sensitivity analysis

We performed sensitivity analyses to determine whether the results were robust to the differences that arise from uncertainties in the parameters. We performed one-way sensitivity analysis to examine the uncertainty surrounding each parameter (e.g., discounting rates, drug efficacy, utility and cost). Except for the discount rates for costs and outcomes, which were set at 0% and 6% per year, the value of a variable was varied from the lower to upper bounds of a confidence interval. Then, the results were illustrated by a tornado diagram. We conducted probabilistic sensitivity analysis to examine the effect of all parameter uncertainties simultaneously using a Monte Carlo simulation function in Microsoft Office Excel 2016. We ran the simulation in 5,000 iterations to yield a range of possible values (i.e., total uncertainties) for the model parameters, which we then incorporated in the estimates of expected costs and QALY, and presented the findings as a cost-effectiveness acceptability curve (CEAC).

**Budget impact analysis.** The budget impact was conducted to estimate the financial burden from the governmental perspective over a five-year period. The analysis incorporated various parameters, including population data systematically classified based on age groups, with a specific emphasis on individuals aged 50 years and older [23]. The incidence data of hepatocellular carcinoma (HCC) and cholangiocarcinoma in Thailand were employed, revealing an annual occurrence of 27,394 cases [24]. Notably, HCC accounted for 50 percent of the overall population [25]. In a study conducted by Somboon K. et al. [26], it was revealed that 11.2% of HCC patients were classified as having Barcelona Clinic Liver Cancer (BCLC) stage C. Consequently, the estimated number of unresectable HCC (uHCC) patients aged 50 years and above in Thailand reached approximately 1,534 individuals per year. Moreover, the accessibility of atezolizumab plus bevacizumab (A+B) was obtained from 15 gastroenterologists with specialized knowledge and experience in the field of liver cancer. This rigorous investigation disclosed that the initial access rate in the first year was at 14.6 percent, with a progressive annual increase of 6.5 percent from the second to the fifth year (Table 2).

**Table 2:** Number of uHCC patients corresponding to atezolizumab+bevacizumab accessibility.

Year	Access rate	uHCC (cases)
**1**	0.15	224
**2**	0.21	324
**3**	0.28	425
**4**	0.34	525
**5**	0.41	625

In terms of budget assessment, the analysis exclusively encompassed direct medical costs: drug cost and expenses associated with treatment procedures and the monitoring of treatment outcomes. These costs were not discounted.

#### Model validation

The validity of our model was assessed through an expert consultation meeting with a consortium of experts in gastroenterology, oncology, and health economics to ensure that the model structure was an appropriate simplification of reality and that all significant aspects were taken into account. The experts also ensured that the parameters and assumptions used in this research were appropriate for the Thai context. An external modeler reviewed the model to confirm that the mathematical calculations and model equations were coded correctly. Additionally, we conducted internal validation to ensure that the model produced logical results in terms of predicted overall survival by extrapolating results beyond observed data. We found that the model predicted a median overall survival of 4 months for those treated with best supportive care which closely matches the survival rate of 4.26 months obtained from studies of Oranratnachai S et al. [17].

#### Ethical approval

This cost-utility analysis relied on parameters derived from the literature, including mean utility data obtained from a co-author’s previously published [20]. No new data were collected from human participants for this study. Therefore, institutional review board (IRB) approval was not sought or required, as the analysis solely utilized secondary data from published literature.

## Results

**Fig 2:**
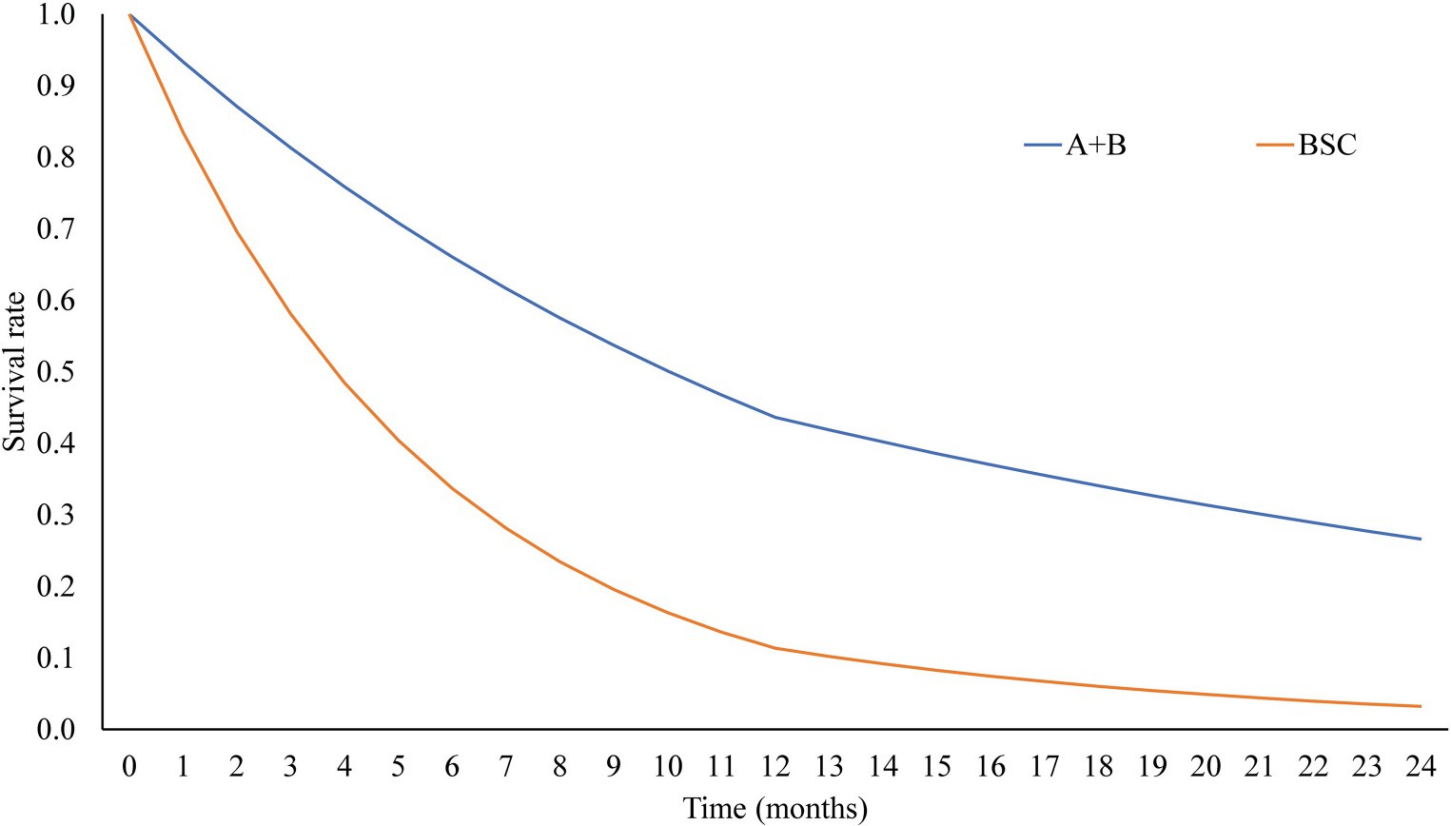
**Predicted overall survival of patients who received atezolizumab plus bevacizumab (A+B) and best supportive care (BSC). Abbreviation:** A+B, atezolizumab plus bevacizumab; BSC, best supportive care.

Our model predicted that patients treated with A+B would have the longest median OS of 10 months compared with those treated with BSC who had the OS of 4 months (Fig 2). In the probabilistic model, A+B treatment had the lifetime cost of $48,669 and outcome of 1.2552 QALYs per person (Table 3). Treatment with BSC had the lifetime cost of $3,313 with the QALY of 0.4243 years. Compared to BSC, the A+B treatment produced the incremental cost of $45,357 and incremental QALY of 0.8309 years, producing an ICER of $54,589 per QALY gained. The drug cost of A+B contributed approximately 85% of total direct medical cost. Moreover, when patients transitioned to a disease progression stage, they would be treated with BSC, the cost of which accounted for only 12% of the total medical cost.

**Table 3:** Incremental cost-effectiveness ratios (ICER, in USD/QALY).

	Atezolizumab+Bevacizumab	BSC
Lifetime cost (USD)		
Direct medical cost	48,363*	3,209
Direct non-medical cost	306	103
Total	48,669	3,312
QALY (Year)	0.8401	0.4051
Incremental cost (USD)	45,357	-
Incremental QALY	0.8309	-
ICER per QALY gained (USD/QALY)	54,589	-

**Abbreviation:** BSC, best supportive care; ICER, incremental cost-effectiveness ratio; QALY, quality-adjusted life year; USD, the United States dollar; *Drug cost of A+B and cost of BSC contributed 85% and 12%, respectively.

**Fig 3:**
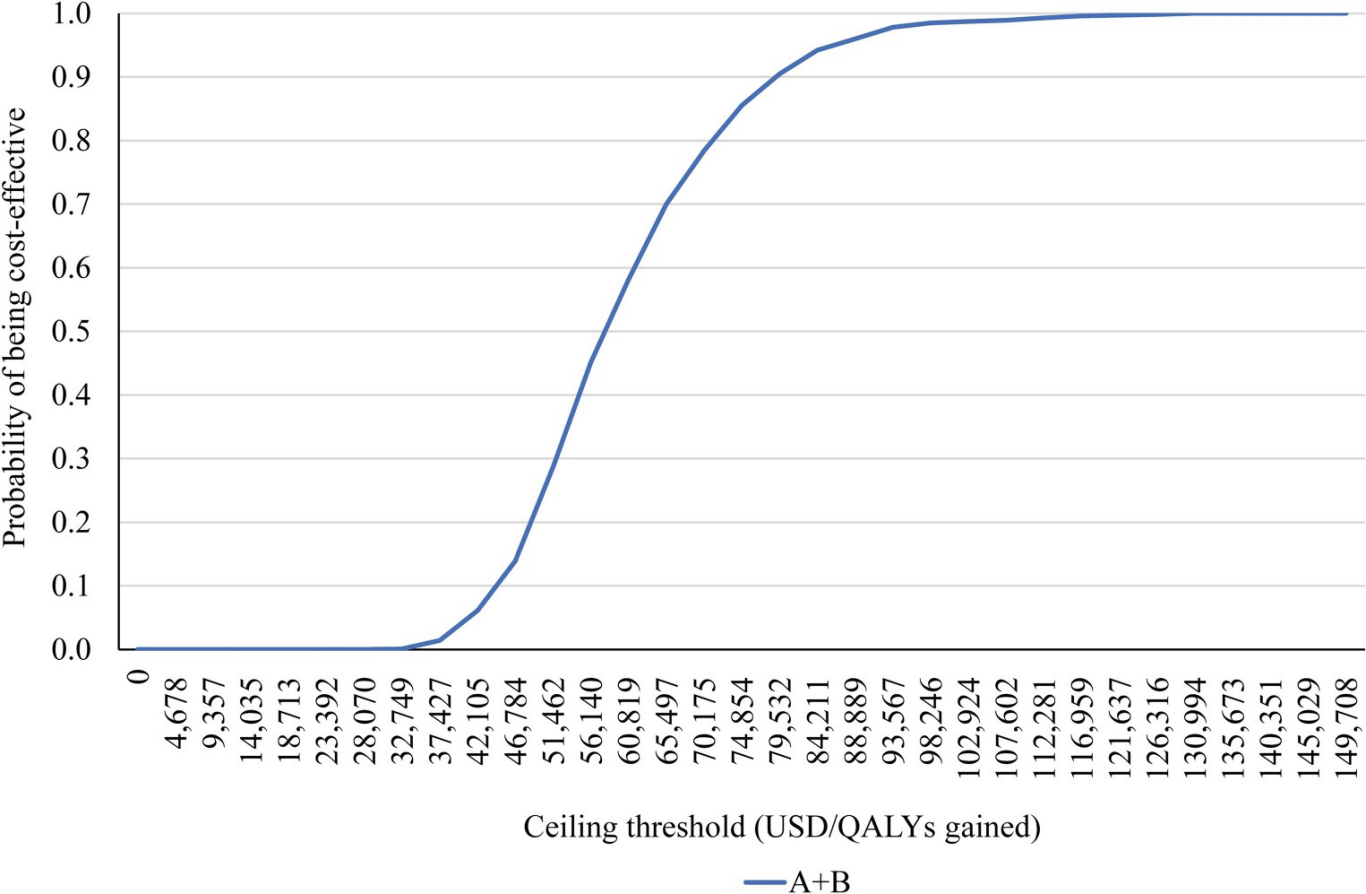
**Cost-effectiveness acceptability curve. Abbreviation:** A+B, atezolizumab plus bevacizumab; QALY, quality-adjusted life year; USD, the United States dollar.

The CEAC after 5,000 simulations showed the relationship between probabilities of each treatment being cost-effective versus the ceiling threshold per additional one QALY. With the Thai ceiling threshold, the probability that A+B was cost-effective for the treatment of uHCC patients was 0% (Fig 3). However, when the ceiling threshold increased to $60,819 per QALY gained, A+B was likely to be the most cost-effective treatment option.

**Fig 4:**
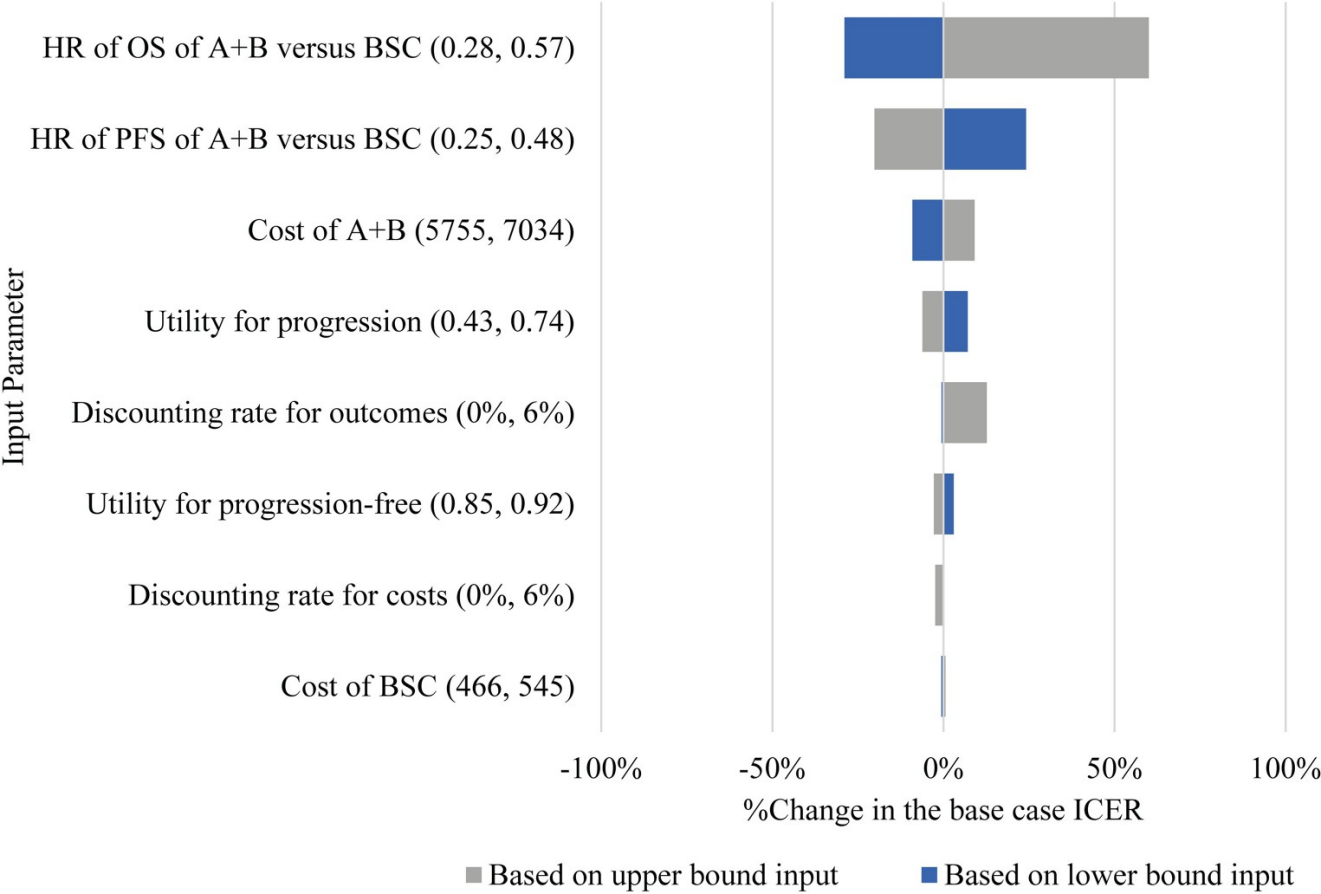
One-way sensitivity analysis on changes in base case ICER for atezolizumab plus bevacizumab (A+B) versus best supportive care (BSC). **Abbreviation:** AB, atezolizumab plus bevacizumab; BSC, best supportive care; CI, confidence interval; ICER, incremental cost-effectiveness ratio; HR, hazard ratio; PFS, progression free survival; OS, overall survival.

The tornado diagram showed parameter sensitivity where the value of a variable varied from the lower to upper bounds of a confidence interval (Fig 4). The most influential variable was the HR for OS of A+B versus BSC, followed by the HR for PFS of A+B versus BSC. However, variations in both parameters did not affect the result that the ICER of A+B versus BSC remained above the ceiling threshold. When the drug cost of A+B is reduced by 6% (to $5,755), the ICER decreases by 9%.

**Fig 5:**
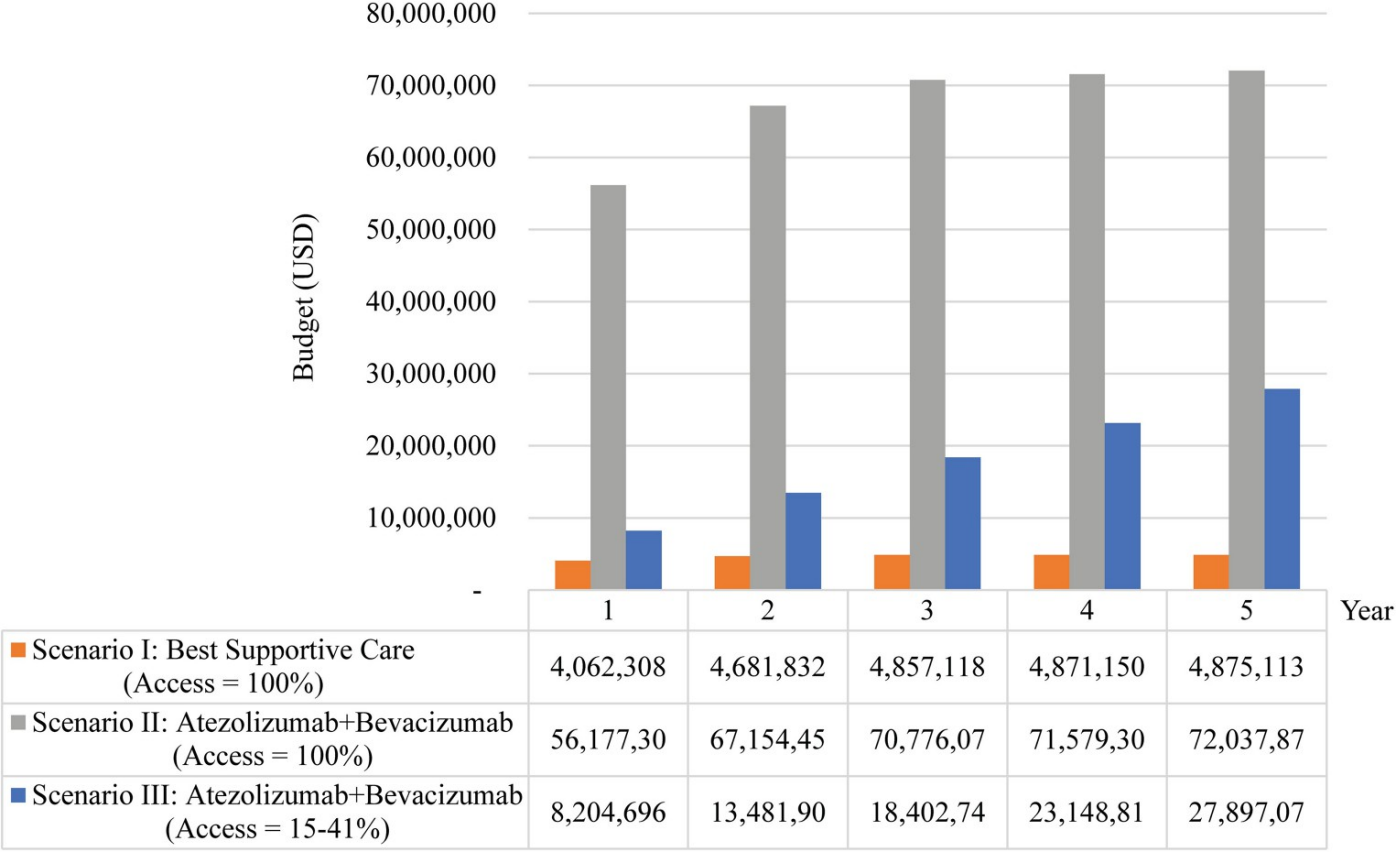
Five-year budget estimation. **Abbreviation:** USD, the United States dollar.

Five-year budget estimation presents the projected budgetary implications resulting from the incorporation of A+B as an alternative therapeutic option for uHCC patients in the national list of essential medicines (NLEM). With an access rate of 15 percent in the first year, followed by rates of 21 percent in the second year, 18 percent in the third year, 34 percent in the fourth year, and 41 percent in the fifth year, the estimated budgetary requirements would range from 8.2 to 27.9 million USD for the respective first and fifth years (Fig 5).

## Discussion

The present study investigated the cost-effectiveness of A+B compared to BSC for treating patients with unresectable hepatocellular carcinoma **(**uHCC**)** in the Thai setting. Our findings showed that A**+**B was clinically superior to BSC, resulting in a 3 times greater quality-adjusted life year (QALY) gained 1.2821 years versus 0.4243 years). However, the lifetime cost of A+B at the current price was 15 times higher than BSC ($48,669 versus $3,312). Consequently, A+B was not cost-effective in the Thai setting at the current threshold of $4,678 per QALY gained. The cost of A+B contributed heavily to the total lifetime cost. Additionally, patients receiving A+B incurred a higher cost for BSC during the disease progression stage compared to the other group, likely due to their prolonged lifespan.

We measured the efficacy of the drug in this study in terms of PFS and OS. We predicted the PFS and OS in patients treated with A+B by using a hazard ratio, where the clinical outcomes of BSC were used as the reference. If the median PFS and median OS were longer in the BSC arm, PFS and OS in patients treated with A+B would increase. In addition, we predicted the median OS of patients treated with A+B in this study to be 10 months, which was inconsistent with the findings from the phase-III trial of A+B (19.2 months) [11]. This can be attributed to the difference in median OS observed in the BSC group, which served as the baseline. We obtained baseline OS data from a study conducted in Thai population [17], which was 0.5 times lower than that reported in the phase-III trial (4.26 months vs 7.90 months).

We found that the result was highly sensitive to the efficacy of A+B. This was because the hazard ratio for OS of A+B versus BSC, which was obtained from a systematic review and network meta-analysis (S1 Appendix), had a wide 95% confidence interval. This contributed to a high level of uncertainty in the model. Our finding from a one-way sensitivity analysis is consisted of previous economic evaluation studies of Zhang X et al. [27] and Chiang et al. [28], which found that efficacy was the parameter with the highest level of uncertainty. Nevertheless, variations in efficacy did not affect the conclusion regarding cost-effectiveness at the current WTP threshold. Furthermore, the probabilistic sensitivity analysis revealed that the finding of A+B was robust. At the current WTP threshold, A+B had 0% chance of being cost-effective. Given Thailand has established the separate WTP threshold ($60,819 per QALY gained) for the treatments of small patient populations which is higher than the ordinary threshold, A+B had a 60% probability of being cost-effective.

Our study results were not comparable with prior cost-effectiveness studies due to differences in the comparators used in the model. Our study used BSC as the comparator, as per the current treatment standards for uHCC in Thailand, while studies in the United States [29] and the Republic of China [27] used sorafenib as a comparison group. However, the findings of this study were in line with other cost-effectiveness studies comparing A+B to other systemic therapies in the United States [29] and Republic of China [27, 28, 30], which found that A+B yielded the highest health benefits but its higher treatment cost negatively affected value for money of A+B in these settings.

Our findings revealed that the cost of A+B was a crucial driving factor of the lifetime cost, however it might be difficult to reach a cost-effective price for A+B by price negotiation alone. Moreover, the one-way sensitivity analysis revealed that uncertainty of the drug’s clinical benefits could potentially affect variations in the ICER. The effectiveness of A+B reported by various observational studies [31–33] was comparable to that of the clinical trial [11]. Likewise, a retrospective study conducted in a single center in Thailand, investigating the efficiency of A+B in treating 83 Thai patients with uHCC from September 2020 to April 2023, yielded a median OS and PFS of 13.0 (95% CI 5.2–20.8) and 9.0 (95% CI 5.0–13.0) months [34]. Patients with Child-Turcotte-Pugh (CTP) class A had a significantly longer median OS compared to those with CTP class B, with values of 17 months and 2.0 months, respectively. This retrospective study also revealed that patients with the modified albumin-bilirubin (mALBI) grade 1 and 2a had significantly longer survival and better clinical outcomes.

The present study assessed the potential budgetary implications that would arise upon the inclusion of the combination therapy A+B as an alternative treatment option for patients with uHCC in the Thailand National List of Essential Medicine. A panel of 15 experts in the field of HCC provided estimates regarding the access rates to systemic therapy for uHCC patients. The analysis projected an initial access rate of 15 percent in the first year, followed by a progressive annual increase of 6.5 percent from the second to the fifth year. Based on these assumptions, it is estimated that there would be a total of 224 uHCC patients with access to systemic therapy in the first year, and this number would increase to 625 patients by the fifth year. Taking into account the prevailing prices of the A+B therapy, the projected budgetary requirements for the respective first and fifth years would range from 8.2 to 27.9 million USD.

In order to enhance access to A+B for Thai patients, alternative financing solutions might be considered such as managed entry agreement (MEA)/risk-sharing agreement (RSA). Generally, financial-based MEA would address budgeting uncertainty and -performance-based MEA could address uncertainty about the performance of technologies. These methods would reduce the overall costs of drug and later improve cost-effectiveness. It is essential for stakeholders to discuss the feasibility of agreement between pharmaceutical companies and payers in the Thai context, since the inclusion of A+B in the National List of Essential Medicine could have a profound impact on public health. Specific patient groups may gain greater benefit from A+B treatment. Apart from Child-Pugh score, the mALBI grade, a simple and validated tool for stratifying liver function in HCC patients, has been shown to predict overall survival and treatment response to A+B [32, 35, 36]. It was found that A+B treatment provided greater clinical efficacy among uHCC patients with mALBI grade 1 or 2a than those with mALBI grade 2b or 3 at baseline [34–36]. Incorporating the modified ALBI score to identify patients who are most likely to benefit from A+B treatment can be a practical approach to optimize cost-effectiveness and budget impact. Real-world evidence (RWE) derived from the Thai setting will prove the predictive performance of the modified ALBI score and provide a more accurate estimate of economic evaluation of A+B.

The strength of this study warrants attention. This cost-effectiveness study utilized recent clinical outcome from Thai patients receiving BSC as the baseline for estimating clinical effects of A+B treatment [17]. The utilization of localized data enhances the reliability of this study within the Thai context. Nevertheless, this study has some limitations. We did not consider compliance and temporary drug discontinuation due to side effects in our analyses.

## Conclusions

The combination of atezolizumab and bevacizumab demonstrated superior efficacy over best supportive care for improving health outcomes of uHCC patients. However, this cost-effectiveness analysis suggested that atezolizumab plus bevacizumab may not be a cost-effective option at current prices. Adopting a practical approach by targeting specific patient groups who are most likely to benefit from A+B treatment, particularly those with favorable the modified albumin-bilirubin (mALBI) grades, could optimize cost-effectiveness and budget impact.

## Supporting information

S1 Appendix Additional parameter information. (DOCX)

## References

[1] World Health Organization: International Agency for Research on Cancer (IARC). Liver: Cancer incidence and mortality statistics worldwide 2020 [updated December 2020; cited 2023 June 28]. Available from: https://gco.iarc.fr/today/data/factsheets/cancers/11-Liver-fact-sheet.pdf.

[2] Janevska D, Chaloska-Ivanova V, Janevski V. Hepatocellular Carcinoma: Risk Factors, Diagnosis and Treatment. Open Access Maced J Med Sci. 2015;3(4):732–6. Epub 2016/06/09. doi: 10.3889/oamjms.2015.111 ; PubMed Central PMCID: PMC4877918. 27275318 PMC4877918

[3] Kuo TM, Chang KM, Cheng TI, Kao KJ. Clinical Factors Predicting Better Survival Outcome for Pulmonary Metastasectomy of Hepatocellular Carcinoma. Liver Cancer. 2017;6(4):297–306. Epub 2017/12/14. doi: 10.1159/000477134 ; PubMed Central PMCID: PMC5704702. 29234633 PMC5704702

[4] Uka K, Aikata H, Takaki S, Shirakawa H, Jeong SC, Yamashina K, et al. Clinical features and prognosis of patients with extrahepatic metastases from hepatocellular carcinoma. World J Gastroenterol. 2007;13(3):414–20. Epub 2007/01/19. doi: 10.3748/wjg.v13.i3.414 ; PubMed Central PMCID: PMC4065897. 17230611 PMC4065897

[5] Katyal S, Oliver JH, Peterson MS, Ferris JV, Carr BS, Baron RL. Extrahepatic metastases of hepatocellular carcinoma. Radiology. 2000;216(3):698–703. Epub 2000/08/31. doi: 10.1148/radiology.216.3.r00se24698 . 10966697

[6] Chan KM, Yu MC, Wu TJ, Lee CF, Chen TC, Lee WC, et al. Efficacy of surgical resection in management of isolated extrahepatic metastases of hepatocellular carcinoma. World J Gastroenterol. 2009;15(43):5481–8. Epub 2009/11/17. doi: 10.3748/wjg.15.5481 ; PubMed Central PMCID: PMC2778106. 19916180 PMC2778106

[7] Parikh ND, Marshall VD, Singal AG, Nathan H, Lok AS, Balkrishnan R, et al. Survival and cost-effectiveness of sorafenib therapy in advanced hepatocellular carcinoma: An analysis of the SEER-Medicare database. Hepatology. 2017;65(1):122–33. Epub 2016/10/23. doi: 10.1002/hep.28881 . 27770556

[8] Vogel A, Meyer T, Sapisochin G, Salem R, Saborowski A. Hepatocellular carcinoma. Lancet. 2022;400(10360):1345–62. Epub 2022/09/10. doi: 10.1016/S0140-6736(22)01200-4 . 36084663

[9] Lu LC, Shao YY, Chan SY, Hsu CH, Cheng AL. Clinical characteristics of advanced hepatocellular carcinoma patients with prolonged survival in the era of anti-angiogenic targeted-therapy. Anticancer Res. 2014;34(2):1047–52. Epub 2014/02/11. . 24511053

[10] Reig M, Forner A, Rimola J, Ferrer-Fàbrega J, Burrel M, Garcia-Criado Á, et al. BCLC strategy for prognosis prediction and treatment recommendation: The 2022 update. J Hepatol. 2022;76(3):681–93. Epub 2021/11/22. doi: 10.1016/j.jhep.2021.11.018 ; PubMed Central PMCID: PMC8866082. 34801630 PMC8866082

[11] Cheng AL, Qin S, Ikeda M, Galle PR, Ducreux M, Kim TY, et al. Updated efficacy and safety data from IMbrave150: Atezolizumab plus bevacizumab vs. sorafenib for unresectable hepatocellular carcinoma. J Hepatol. 2022;76(4):862–73. Epub 2021/12/14. doi: 10.1016/j.jhep.2021.11.030 . 34902530

[12] Working Group for the Development of Health Technology Assessment Guidelines for Thailand (2021). Guidelines for Health Technology Assessment in Thailand, Revised Edition BE 2564. Nonthaburi, Thailand: Health Intervention and Technology Assessment Program; 2021. Available from: https://www.hitap.net/wp-content/uploads/2023/07/Completed_HTA_Guide2564_260923.pdf.

[13] Working Group for the Development of Health Technology Assessment Guidelines for Thailand. Guidelines for Health Technology Assessment in Thailand. 1st ed. Nonthaburi, Thailand: The Graphico Systems Co., Ltd; 2009. Available from: https://www.hitap.net/documents/20711.

[14] Sonbol MB, Riaz IB, Naqvi SAA, Almquist DR, Mina S, Almasri J, et al. Systemic Therapy and Sequencing Options in Advanced Hepatocellular Carcinoma: A Systematic Review and Network Meta-analysis. JAMA Oncol. 2020;6(12):e204930. Epub 2020/10/23. doi: 10.1001/jamaoncol.2020.4930 ; PubMed Central PMCID: PMC7582230 33090186 PMC7582230

[15] Llovet JM, Ricci S, Mazzaferro V, Hilgard P, Gane E, Blanc JF, et al. Sorafenib in advanced hepatocellular carcinoma. N Engl J Med. 2008;359(4):378–90. Epub 2008/07/25. doi: 10.1056/NEJMoa0708857 . 18650514

[16] Cheng AL, Kang YK, Chen Z, Tsao CJ, Qin S, Kim JS, et al. Efficacy and safety of sorafenib in patients in the Asia-Pacific region with advanced hepatocellular carcinoma: a phase III randomised, double-blind, placebo-controlled trial. Lancet Oncol. 2009;10(1):25–34. Epub 2008/12/20. doi: 10.1016/S1470-2045(08)70285-7 . 19095497

[17] Oranratnachai S, Rattanasiri S, Sirachainan E, Tansawet A, Raunroadroong N, McKay GJ, et al. Treatment outcomes of advanced hepatocellular carcinoma in real‐life practice: Chemotherapy versus multikinase inhibitors. 2023;12(3):3046–53. doi: 10.1002/cam4.5224 36082831 PMC9939209

[18] Thongsawat S, Piratvisuth T, Pramoolsinsap C, Chutaputti A, Tanwandee T, Thongsuk D. Resource Utilization and Direct Medical Costs of Chronic Hepatitis C in Thailand: A Heavy but Manageable Economic Burden. Value Health Reg Issues. 2014;3:12–8. Epub 2014/05/01. doi: 10.1016/j.vhri.2013.09.002 . 29702917

[19] Riewpaiboon A. Standard Cost List for Health Technology Assessment Nonthaburi, Thailand: Health Intervention and Technology Assessment Program (HITAP), Ministry of Public Health; 2009. Available from: http://costingmenu.hitap.net/.

[20] Charonpongsuntorn C, Tanasanvimon S, Korphaisarn K, Payapwattanawong S, Siripoon T, Pakvisal N, et al. Efficacy, safety, and patient-reported outcomes of atezolizumab plus bevacizumab for unresectable hepatocellular carcinoma in Thailand: A multicenter prospective study. 2022;8:e2200205. doi: 10.1200/GO.22.00205 36455172 PMC10166432

[21] World Health Organization. Global Health Observatory data repository: Life tables by country (Thailand) 2020 [updated December 16, 2020; cited 2023 June 28]. Available from: https://apps.who.int/gho/data/?theme=main&vid=61640.

[22] Bureau of Trade and Economic Indices, Ministry of Commerce. Report for consumer price index of Thailand year 2021 base year 2019 2021 [cited 2023 June 28]. Available from: http://www.price.moc.go.th/price/cpi/index_new_all.asp.

[23] Ministry of Interior, Thailand. Population classified by age groups. Available from: http://stat.dopa.go.th/stat/statnew/upstat_age.php.

[24] World Health Organization. Incidence, Mortality and Prevalence by cancer site 2021. Available from: https://gco.iarc.fr/today/data/factsheets/populations/764-thailand-fact-sheets.pdf.

[25] Maneeon S, Chaikledkaew U, Teerawattananon Y. Cost-effectiveness analysis of Lipiodol Ultra Fluid injection for the treatment of hepatocellular carcinoma 2014.

[26] Somboon K, Siramolpiwat S, Vilaichone RK. Epidemiology and survival of hepatocellular carcinoma in the central region of Thailand. Asian Pac J Cancer Prev. 2014;15(8):3567–70. Epub 2014/05/30. doi: 10.7314/apjcp.2014.15.8.3567 . 24870758

[27] Zhang X, Wang J, Shi J, Jia X, Dang S, Wang W. Cost-effectiveness of Atezolizumab Plus Bevacizumab vs Sorafenib for Patients With Unresectable or Metastatic Hepatocellular Carcinoma. JAMA Network Open. 2021;4(4):e214846-e. doi: 10.1001/jamanetworkopen.2021.4846 33825837 PMC8027915

[28] Chiang CL, Chan SK, Lee SF, Choi HC. First-Line Atezolizumab Plus Bevacizumab versus Sorafenib in Hepatocellular Carcinoma: A Cost-Effectiveness Analysis. Cancers (Basel). 2021;13(5). Epub 2021/03/07. doi: 10.3390/cancers13050931 ; PubMed Central PMCID: PMC7956424. 33668100 PMC7956424

[29] Patel K, Stein S, Luther J, Huntington SF. Cost-effectiveness of atezolizumab and bevacizumab in advanced hepatocellular carcinoma. Journal of Clinical Oncology. 2021;39(15_suppl):e18829-e. doi: 10.1200/JCO.2021.39.15_suppl.e18829

[30] Su D, Wu B, Shi L. Cost-effectiveness of Atezolizumab Plus Bevacizumab vs Sorafenib as First-Line Treatment of Unresectable Hepatocellular Carcinoma. JAMA Netw Open. 2021;4(2):e210037. Epub 2021/02/25. doi: 10.1001/jamanetworkopen.2021.0037 ; PubMed Central PMCID: PMC7905498. 33625508 PMC7905498

[31] Himmelsbach V, Pinter M, Scheiner B, Venerito M, Sinner F, Zimpel C, et al. Efficacy and Safety of Atezolizumab and Bevacizumab in the Real-World Treatment of Advanced Hepatocellular Carcinoma: Experience from Four Tertiary Centers. Cancers (Basel). 2022;14(7). Epub 2022/04/13. doi: 10.3390/cancers14071722 ; PubMed Central PMCID: PMC8996828. 35406493 PMC8996828

[32] Fulgenzi CAM, Cheon J, D’Alessio A, Nishida N, Ang C, Marron TU, et al. Reproducible safety and efficacy of atezolizumab plus bevacizumab for HCC in clinical practice: Results of the AB-real study. European Journal of Cancer. 2022;175:204–13. doi: 10.1016/j.ejca.2022.08.024 36148739

[33] de Castro T, Jochheim LS, Bathon M, Welland S, Scheiner B, Shmanko K, et al. Atezolizumab and bevacizumab in patients with advanced hepatocellular carcinoma with impaired liver function and prior systemic therapy: a real-world experience. Ther Adv Med Oncol. 2022;14:17588359221080298. Epub 2022/03/08. doi: 10.1177/17588359221080298 ; PubMed Central PMCID: PMC8891886. 35251317 PMC8891886

[34] Navadurong H, Prasoppokakorn T, Siriwong N, Phathong C, Teeyapun N, Tanasanvimon S, et al. Modified albumin-bilirubin predicted survival of unresectable hepatocellular carcinoma patients treated with immunotherapy. World J Gastrointest Oncol. 2023;15(10):1771–83. doi: 10.4251/wjgo.v15.i10.1771 37969413 PMC10631433

[35] Kudo M, Finn RS, Cheng A-L, Zhu AX, Ducreux M, Galle P, et al., editors. IMbrave150: albumin-bilirubin (ALBI) grade analyses in a phase III study of atezolizumab (atezo) + bevacizumab (bev) vs sorafenib (sor) in patients (pts) with unresectable hepatocellular carcinoma (HCC). the 15th International Liver Cancer Association Conference; 2021 September 2–5, 2021; Virtual Conference.

[36] Tomonari T, Tani J, Sato Y, Tanaka H, Tanaka T, Taniguchi T, et al. Initial therapeutic results of atezolizumab plus bevacizumab for unresectable advanced hepatocellular carcinoma and the importance of hepatic functional reserve. Cancer Med. 2023;12(3):2646–57. Epub 2022/08/15. doi: 10.1002/cam4.5145 ; PubMed Central PMCID: PMC9939118. 35964253 PMC9939118

