# Supplementary material for: Cost-utility analysis of atezolizumab combined with bevacizumab for unresectable hepatocellular carcinoma in Thailand
Source: PLoS One. 2024 Mar 21;19(3):e0300327. doi: 10.1371/journal.pone.0300327 (PMC10956810; doi:10.1371/journal.pone.0300327)
Supplement: S1 Appendix — (DOCX) [file pone.0300327.s001.docx]

**Supporting information**

**Cost-utility analysis of atezolizumab combined with bevacizumab for unresectable hepatocellular carcinoma in Thailand**

PLOS ONE

[Supachaya Sriphoosanaphan](https://pubmed.ncbi.nlm.nih.gov/?term=Sriphoosanaphan+S&cauthor_id=33614272)^¶^, Witthawat Pantumongkol^¶^, Wantanee Kulpeng, Chanchai Charonpongsuntorn, Tawesak Tanwandee, Wattana Sukeepaisarnjaroen, Abhasnee Sobhonslidsuk, Pisit Tangkijvanich^*^

* Corresponding author

Email: pisittkvn@yahoo.com [PT]

^¶^These authors contributed equally to this work.

**S1 Appendix. Additional parameter information**

**1. The mortality rate of the Thai general population**

**Table S1: All causes mortality of Thai population classified by age range**

| **Age group (year)** | **Probability (Mean)** | **Standard error** |
| --- | --- | --- |
| 50-54 | 0.006178 | 0.000035 |
| 55-59 | 0.008413 | 0.000043 |
| 60-64 | 0.012056 | 0.000058 |
| 65-69 | 0.017150 | 0.000078 |
| 70-74 | 0.050713 | 0.000198 |
| 75-79 | 0.050713 | 0.000198 |
| 80-84 | 0.050713 | 0.000198 |
| 85-89 | 0.050713 | 0.000198 |
| 90-94 | 0.150000 | 0.000198 |
| 95-99 | 0.200000 | 0.000198 |
| 100 years and above+ | 0.632121 | 0.000198 |

**Source:** Strategy and Planning Division, Ministry of Public Health. Mortality rate by age and gender per 1,000 Thai people in 2023.

**2. Network meta-analysis**

In Thailand, best supportive care has been a standard of care for majority of Thai people as all systemic therapies, including immunotherapy are not reimbursable. However, there was no head-to-head trial comparing the combination of atezolizumab and bevacizumab with best supportive care. Therefore, indirect comparison via sorafenib was conducted to obtain efficacy parameters for this study. Prior to the indirect comparison, systematic review and pooling estimates from randomized control trials (RCTs) examining atezolizumab and bevacizumab versus best supportive care or placebo were performed.

We evaluated potentially eligible RCTs identified by the recent systematic review of systemic therapies in advanced hepatocellular carcinoma (HCC) was published in 2020 [1]. Only 3 RCTs were included in our analysis. The network meta-analysis was carried out by using the R software with the package “Netmeta”. The random effects model was applied. Results from network meta-analysis showed that atezolizumab and bevacizumab was associated with the longer time to progression than best supportive care or placebo (HR = 0.34, 95%CI 0.25 to 0.46) and as well as the higher benefit in terms of overall survival (HR = 0.40, 95%CI 0.28 to 0.57).

**
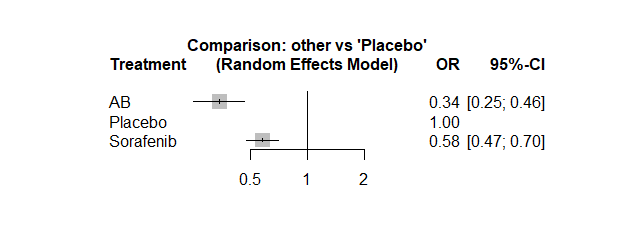
**

**Fig S1.** **Progression free survival (PFS)**


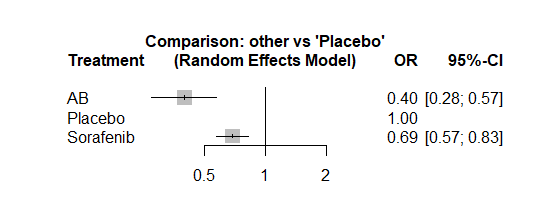


**Fig S2. Overall survival (OS)**

**3. Adverse event**

Only the major grade 3 to 4 adverse event (AE) with an occurrence rate of more than 5% are selected as agreed upon in the expert consultation meeting. AEs are extracted from IMbrave150 trial [2]. Resource used for treatment-related serious AEs are obtained from HCC experts and unit costs are elicited from DMSIC, Thai FDA.

**Table S2:** Grade 3 or 4 AEs related cost

| **Major AEs** | **Percentage** | **Resource used** | **Unit cost** |
| --- | --- | --- | --- |
| Hypertension | 15.2% | Amlodipine 10 mg once daily | Amlodipine 10 mg ($0.04 per tablet). |
| Aspartate aminotransferase increase | 7.0% | Dose reduction | - |

**4. Utility data**

In order to calculate mean utility data specific to our health states, we proactively contacted the corresponding author of a pertinent local study [3]. The health states considered were those corresponding to the pre- and post-progression health states, and the utility data was extracted from their dataset. For those interested in accessing the raw utility data used in this study, please feel free to contact:

Chanchai Charonpongsuntorn, MD

Division of Medical Oncology, Department of Internal Medicine,

Faculty of Medicine, Srinakharinwirot University,

62 Moo 7 Ongkharak, Nakhon Nayok 26120, Thailand

Email: chanchaic@g.swu.ac.th.

**References:**

1. Sonbol MB, Riaz IB, Naqvi SAA, Almquist DR, Mina S, Almasri J, et al. Systemic Therapy and Sequencing Options in Advanced Hepatocellular Carcinoma: A Systematic Review and Network Meta-analysis. JAMA Oncol. 2020;6(12):e204930. Epub 2020/10/23. doi: 10.1001/jamaoncol.2020.4930. PubMed PMID: 33090186; PubMed Central PMCID: PMCPMC7582230

2. Finn RS, Qin S, Ikeda M, Galle PR, Ducreux M, Kim T-Y, et al. Atezolizumab plus bevacizumab in unresectable hepatocellular carcinoma. 2020;382(20):1894-905.

3. Charonpongsuntorn C, Tanasanvimon S, Korphaisarn K, Payapwattanawong S, Siripoon T, Pakvisal N, et al. Efficacy, safety, and patient-reported outcomes of atezolizumab plus bevacizumab for unresectable hepatocellular carcinoma in Thailand: A multicenter prospective study. 2022;8:e2200205.
